# The PECACE domain: a new family of enzymes with potential peptidoglycan cleavage activity in Gram-positive bacteria

**DOI:** 10.1186/1471-2164-6-19

**Published:** 2005-02-17

**Authors:** Estelle Pagliero, Otto Dideberg, Thierry Vernet, Anne Marie Di Guilmi

**Affiliations:** 1 Laboratoire d'Ingénierie des Macromolécules Institute de Biologie Structurale Jean-Pierre Ebel (CEA-CNRS UMR 5075-UJF), 41 Rue Jules Horowitz 38027 Grenoble cedex 1, France; 2 Laboratoire de Cristallographie Macromoléculaire Institut de Biologie Structurale Jean-Pierre Ebel (CEA-CNRS UMR 5075-UJF), 41 Rue Jules Horowitz 38027 Grenoble cedex 1, France

## Abstract

**Background:**

The metabolism of bacterial peptidoglycan is a dynamic process, synthases and cleavage enzymes are functionally coordinated. Lytic Transglycosylase enzymes (LT) are part of multienzyme complexes which regulate bacterial division and elongation. LTs are also involved in peptidoglycan turnover and in macromolecular transport systems. Despite their central importance, no LTs have been identified in the human pathogen *Streptococcus pneumoniae*. We report the identification of the first putative LT enzyme in *S. pneumoniae *and discuss its role in pneumococcal peptidoglycan metabolism.

**Results:**

Homology searches of the pneumococcal genome allowed the identification of a new domain putatively involved in peptidoglycan cleavage (PECACE, PEptidoglycan CArbohydrate Cleavage Enzyme). This sequence has been found exclusively in Gram-positive bacteria and gene clusters containing *pecace *are conserved among Streptococcal species. The PECACE domain is, in some instances, found in association with other domains known to catalyze peptidoglycan hydrolysis.

**Conclusions:**

A new domain, PECACE, putatively involved in peptidoglycan hydrolysis has been identified in *S. pneumoniae*. The probable enzymatic activity deduced from the detailed analysis of the amino acid sequence suggests that the PECACE domain may proceed through a LT-type or goose lyzosyme-type cleavage mechanism. The PECACE function may differ largely from the other hydrolases already identified in the pneumococcus: LytA, LytB, LytC, CBPD and PcsB. The multimodular architecture of proteins containing the PECACE domain is another example of the many activities harbored by peptidoglycan hydrolases, which is probably required for the regulation of peptidoglycan metabolism. The release of new bacterial genomes sequences will probably add new members to the five groups identified so far in this work, and new groups could also emerge. Conversely, the functional characterization of the unknown domains mentioned in this work can now become easier, since bacterial peptidoglycan is proposed to be the substrate.

## Background

**Figure 1:**
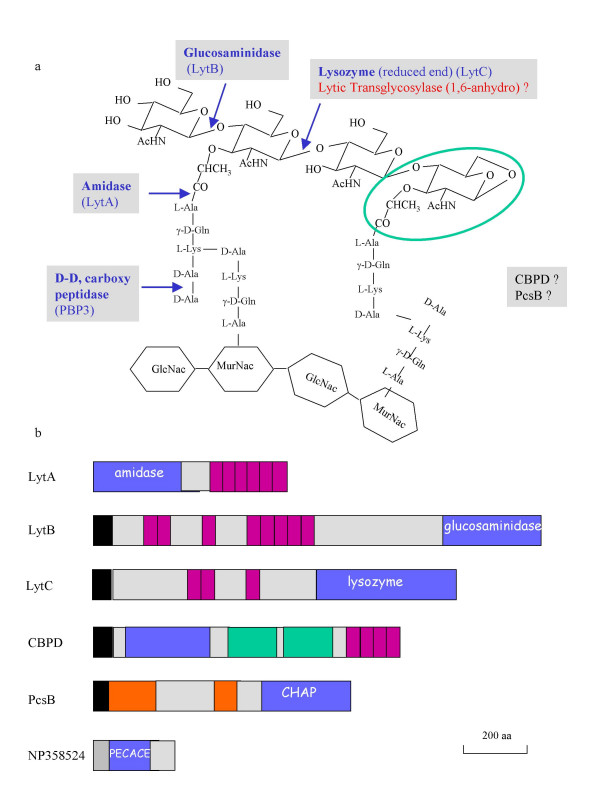
**Schematic representation of peptidoglycan and of cleavage enzymes in *S. pneumoniae*. **(a) Scheme of the pneumococcal peptidoglycan, indicating the chemical bonds cleaved by identified hydrolases in blue. The Mur*N*Ac residue containing the 1, 6-anhydro bond resulting from LT reaction is in a green circle. The putative LT pneumococcal enzyme appears in red, while enzymes CBPD and PcsB for which no enzymatic specificity is yet characterized are in black. (b) Topological representation of the glycan strand hydrolases described in *S. pneumoniae*. Black and hatched boxes indicate the signal peptide and the transmembrane anchor, respectively. The blue boxes illustrate the respective enzymatic active domains. Purple rectangles correspond to the Choline-Binding repeats. Green and orange boxes correspond to SH3b and coiled-coil regions, respectively. The topology was designed with the help of SMART server [39].

The bacterial cell wall resists intracellular pressure and gives the bacterium its particular shape. Cell wall reinforcement is brought about by a strong scaffolding structure, the peptidoglycan, which is formed by glycan strands and peptide chains held together by covalent bonds, resulting in a mono- or multilayered network. The glycan strands are composed of *N*-acetylglucosamine (Glc*N*Ac) and *N*-acetylmuramyl (Mur*N*Ac) residues linked together by β-1,4 glycosidic bonds. Peptides are covalently attached to the lactyl group of the muramic acid and their cross-linking results in the net structure of the peptidoglycan (Fig. 1a).

Peptidoglycan is synthesized in a multi-stage process. The first steps occur in the cytoplasm, where a set of enzymatic reactions gives rise to the assembly of the Mur*N*Ac-pentapeptide. This unit is in turn linked to the carrier undecaprenol lipid via a pyrophosphate group; afterwards the Glc*N*Ac group is added, generating the lipid II precursor. The saccharidic and peptidic moieties of lipid II are subsequently exposed to the periplasmic space. At this stage, peptidoglycan biosynthesis involves polymerization of the glycan chains, catalyzed by glycosyltransferases [1] as well as interpeptide bridge formation performed by transpeptidases [2]. These two enzymatic reactions are resident on the extracellular domains of Penicillin-Binding Proteins (PBPs) which are membrane-associated molecules, present in all eubacteria [2].

Peptidoglycan metabolism is a dynamic process since this structure grows and divides in perfect synchronization with cell growth and division. Furthermore, it is well established that peptidoglycan is subject to maturation, turnover and recycling in Gram-negative bacteria [3]. To fullfil these processes, it is expected that peptidoglycan cleavage enzymes must exert their functions in coordinated action with PBPs. Indeed, a large range of different peptidoglycan hydrolases have been identified in numerous bacterial species and specific peptidoglycan hydrolases exist for almost each covalent bond [3] (Fig. 1a). The polysaccharidic component of peptidoglycan is the target of several hydrolases: the β-1,4 glycosidic bond between Mur*N*Ac and Glc*N*Ac residues is cleaved by lyzosyme and by lytic transglycosylases (LT), the β-1,4 glycosidic bond between Glc*N*Ac and Mur*N*Ac is hydrolyzed by glucosaminidases and amidases are responsible for the cleavage of the Mur*N*Ac-L-alanine bond (Fig. 1a).

Lyzosyme and LT enzymes cleave the same β-1,4-Mur*N*Ac-GlcNAc bond but generate different reaction products: while lyzosymes catalyze a hydrolytic reaction, LTs cleave the β-glycosidic linkage with the concomitant formation of 1,6-anhydromuramyl residues, blocking the reducing end of the glycan strands. The significance of the ring structure is not known but it has been speculated that the bond energy may be utilized for glycan strand rearrangements. In addition, the 1,6-anhydro ring may also be considered as a specific product of peptidoglycan turnover. Despite the lack of understanding of the physiological function of anhydromuropeptide product, LT enzymes must play a significant cellular role. Indeed, it has been observed that deletions of genes encoding LT proteins lead to *E. coli *and *Neisseria meningitidis *with altered cell separation phenotypes, indicating that LTs cleave septal peptidoglycan [4,5]. Macromolecular transport systems (secretion types II, III, IV and IV pilus synthesis) of Gram-negative bacteria contain LT enzymes, suggesting that peptidoglycan hole formation (essential for transport functions) is specifically performed by this enzyme family [6]. As mentioned above, the enlargement of the bacterial stress-bearing peptidoglycan structure requires the well coordinated action of synthases (PBPs) and hydrolase enzymes. The "three-for-one" growth mechanism described by Höltje proposes that a triplet of glycan strands cross-linked to each other (resulting from PBPs synthesis) is attached to the peptidoglycan layer. Subsequently, the docking strand is removed by hydrolases resulting in the insertion of the peptidoglycan triplet. The hydrolases involved in such multienzyme complexes are endopeptidases and LT enzymes [3]. This hypothesis is supported by experimental data as LT and PBPs could be co-purified from *E. coli *extracts [7-9]. In conclusion, LT enzymes play an important cellular role in diverse aspects of cell biology as expected from their presence in a very wide range of eubacteria as well as archaebacteria [3,10,11].

Surprinsingly, no such LT enzyme has been identified to date in the human pathogen *Streptococcus pneumoniae*, the causative agent of ear infections in children, as well as meningitis and pneumonia. The pattern of peptidoglycan hydrolases in this Gram-positive bacteria includes, besides a D, D-carboxypeptidase, five glycan strand cleaving enzymes (Fig 1b). Four of these are surface-exposed proteins harboring Choline-Binding Domains which are non-covalently bound to choline residues present on cell wall pneumococcal teichoic and lipoteichoic acids [12-14]. The Choline-Binding Proteins (CBPs) catalyzing peptidoglycan hydrolysis are LytA, LytB, LytC and potentially CBPD (Fig 1b). LytA is an amidase and also appears as an autolytic enzyme, causing bacteriolysis when acting in an uncontrolled manner [15]. LytB is a glucosaminidase involved in cell separation as *lytB *mutants form very long chains of over 100 cells [16]. LytC is a lysozyme with an autolytic behavior at 30°C [17]. Finally, CBPD and PcsB contain a CHAP domain (Cysteine, Histidine-dependent amidohydrolase/peptidase) predicted to hydrolyse the peptidoglycan in pneumococcus, but definitive biochemical data are still lacking [18-20].

Our interest in the biology of *S. pneumoniae *led us to investigate the presence of LT enzymes in this bacteria. Homology searches of enzyme sequences within the pneumococcus genome using bioinformatics tools allowed the identification of a new domain harboring motifs that infer potential peptidoglycan cleavage activity. For this reason we named this domain PECACE (PEptidoglycan CArbohydrate Cleavage Enzyme). This domain sequence was found exclusively in Gram-positive bacterial species, suggesting a significant cellular role. Finally, the PECACE domain is in some instances found in association with other domains, known to catalyze peptidoglycan hydrolysis: this observation reinforces the predicted function of PECACE as participating in peptidoglycan cleavage and represents another example of multifunctional proteins involved in peptidoglycan metabolism.

## Results and discussion

### Identification of a protein harboring the PECACE domain in *S. pneumoniae*

The C-terminal domain of *Escherichia coli *Slt70 (Soluble Lytic Transglycosylase) has a lysozyme-like fold and its amino acid sequence was employed in a search of Bacilli genomes within the NCBI Conserved Domain Search server [11,21-23]. Thirty-four Slt70-homologue sequences were retrieved using an inclusion threshold of 0.01. None of these sequence originated from the *S. pneumoniae *translated genome. Subsequently, each of these 34 sequences was compared with the non-redundant protein database using PSI-BLAST with a E-value threshold of 0.005 and 5 sequences showed significant matches with a unique protein in *S. pneumoniae*. This sequence (accession numbers NP358524, gi:15902974) contains 204 amino acids: the first 21 amino acids are predicted to form a transmembrane anchor and the subsequent 192-residue region is putatively exposed to the extracellular space (Fig. 1b). This *S. pneumoniae *NP358524 sequence has been tested as a pneumococcal vaccine antigen on the basis of preliminary screens for novel vaccine candidates [24].

**Figure 2:**
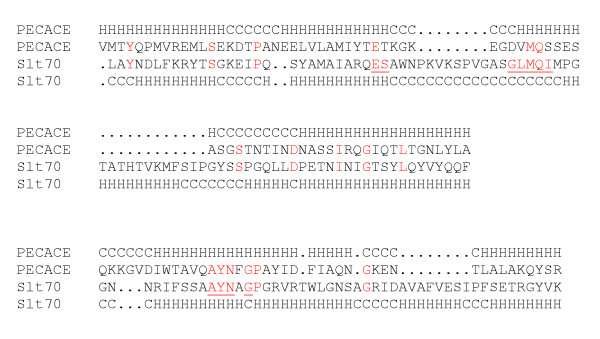
**Alignment of the PECACE domain with Slt70. **Protein fold recognition was performed with the 3D-PSSM server. The NP358524 sequence (residues 31–145) from *S. pneumoniae *(PECACE domain) is aligned with Slt70 from *E. coli *(P03810, residues 478–616). Amino acids of Slt70 involved in the catalytic reaction and in ligand recognition are underlined while residues conserved in each alignment are highlighted in red. The structural prediction for *S. pneumoniae *PECACE domain was determined (H = helix, C = coil) while Slt70 secondary-structure information was obtained from PDB file 1QSA.

A three-dimensional fold prediction of the *S. pneumoniae *NP358524 protein was performed with the 3D-PSSM server [25] which identified two matches: *E. coli *Slt70 (d1qsaa2, E-value:10^-7^) and LysG (G-type goose lyzozyme, d1531, E-value:10^-3^). The sequence alignment between NP358524 and Slt70 is shown in Fig. 2, defining the PECACE domain in the pneumococcal protein. The secondary structures are also reported, based on three-dimensional structures of Slt70 and on computational predictions for PECACE and suggest that the latter is highly α-helical (Fig. 2). It is of note that both Slt70 and LysG are highly similar, and both lack the catalytic aspartate residue commonly found in the active site of lysozymes [10,11,21,22]. Therefore, the PECACE domain of the NP358524 sequence appears to belong to this group of bacterial lysozymes, characterized by the absence of an aspartate residue in the catalytic site and is part of the Glycoside Hydrolase family 23 based upon CAZy classification . The catalytic acid residue in the PECACE domain is most probably Glu61 since it aligns with the catalytic Glu478 residue in the Slt70 sequence (Fig. 2). The serine residue following the catalytic glutamate and the GLMQI/V motif are essential for active-site architecture and are conserved between Slt70 and LysG. In the PECACE sequence, a threonine residue follows the catalytic glutamate and the GLMQI/V motif differs since the corresponding sequence is D(68)VMQS (Fig. 2). Finally, the second motif AYNxG which has been shown to be involved in the interaction with the substrate for Slt70 (A551YNxG) is well conserved in the PECACE sequence (A117YNxG).

Based on this sequence analysis, we infer that the *S. pneumoniae *NP358524 protein, through its PECACE domain, probably catalyzes the peptidoglycan cleavage of the β-1,4-Mur*N*Ac-Glc*N*Ac bond by employing Glu61 as the catalytic residue.

### Identification of the PECACE domain in Gram-positive bacteria

**Figure 3:**
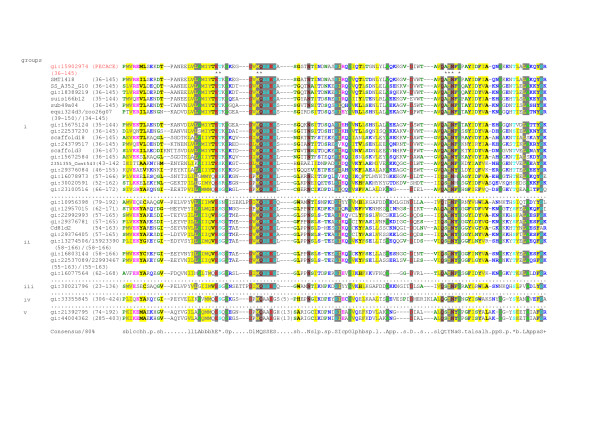
**Sequence alignment of PECACE domains identified in Gram-positive bacteria**. Multiple sequence alignment was constructed using ClustalW. The lengths of the insertions in the sequences are shown in parentheses. The sequences are denoted by their GenBank Identifier (gi). The domain limits are indicated by the residue positions (first-end). The amino acids identified as catalytic or involved in ligand recognition are marked with asterisks under PECACE sequence. Alignments are coloured using the CHROMA tool using default parameters [40]. Full sequence details, group (i): *Streptococcus pneumoniae R6 *(gi:15902974), *Streptococcus mitis NCTC 12261 *(§SMT1418), *Streptococcus sanguinis SK36 *(&:SS_A352_G10), *Streptococcus gordonii *(gi:18389219), *Streptococcus suis P1/7 *(suis166b12), *Streptococcus uberis 0140J *(sub49a04), *Streptococcus equi *(equi324d3), *Streptococcus equi subsp. Zooepidemicus *(zoo26g07), *Streptococcus pyogenes M1 GAS *(gi:15675124), *Streptococcus agalactiae 2603V/R *(gi:22537230), *Lactococcus lactis subsp. Cremoris SK11 *(scaffold18), *Streptococcus mutans UA159 *(gi:24379517), *Streptococcus thermophilus LMD-9 *(scaffold3), *Lactococcus lactis subsp. Lactis *(gi:15672584), *Enterococcus faecium DO *(2351355_Cont543), *Enterococcus faecalis V583 *(gi:29376084), *Bacillus subtilis subsp. subtilis str. 168 *(gi:16078973), *Bacillus cereus ATCC 14579 *(gi:30020591), *Oceanobacillus iheyensis HTE831 *(gi:23100516), group (ii): *Bacillus anthracis*: (pXO2-08) (gi:10956398), *Enterococcus faecalis*: (pRE25) (gi:12957015), *Enterococcus faecium *(gi:22992993), *Enterococcus faecalis V583 *(gi:29376781), *Clostridium difficile 630 *(Cd81d2), *Enterococcus faecalis V583 *(gi:29376405), *Clostridium perfringens *(gi:13274506), *Staphylococcus aureus subsp. aureus Mu50 *(gi:15923390), *Listeria monocytogenes EGD-e *(gi:16803144), *Streptococcus agalactiae 2603V/R *(gi:22537089), *Enterococcus faecium *(gi:22993467), *Bacillus subtilis subsp. subtilis str. 168 *(gi:16077564, group (iii): *Bacillus cereus ATCC 14579 *(gi:30021796), group (iv): *Enterococcus faecalis BM4518 *(gi:33355845), group (v): *Bacillus anthracis *str. A2012: (pXO1) (gi:21392795), *Bacillus cereus ATCC 10987*: (pBc10987) (gi:44004362).

The 204 amino acid sequence from *S. pneumoniae *NP358524, containing the PECACE domain, was used as a PSI-BLAST search query. In total, 29 distinct proteins, all from Gram-positive bacteria, were identified (E-value: 10^-5^) and no sequences from Gram-negative bacteria were retrieved. These sequences were aligned with ClustalW and manually edited. A conserved pattern could be extracted from this alignment: E- [ST]-X-G-X(1,16)-D-X-M-Q- [SA]- [SA]-E- [SG] which was used to search for additional sequences, but no new sequence could be detected from databases, even with a degenerated pattern. PSI-BLAST performed through the GOLD server led to the identification of 10 new sequences from Gram-positive bacteria [26]. In summary, out of the about 50 Gram-positive bacteria for which the whole genome sequence is available, 34 of them contain at least one protein harboring the PECACE domain. The final alignment of these sequences with the *S. pneumoniae *PECACE domain is shown in Fig. 3. The putative catalytic glutamate residue, Glu61 in the *S. pneumoniae *PECACE domain, is conserved in all sequences and the following residue is a Ser or Thr in accordance with Slt70 and LysG patterns. In addition, the D(68)VMQS motif in the *S. pneumoniae *PECACE domain is also well represented in the large majority of sequences with the consensus sequence DI/VMQSSES. Finally, the second motif AYNxG is also conserved while the Ala residue is often replaced by a Ser. In conclusion, the features identified in the *S. pneumoniae *PECACE domain regarding the potential enzymatic properties of peptidoglycan polysaccharide cleavage are also shared by the similar PECACE domains in Gram-positive bacteria.

### Genomic organization of *pecace *genes

**Figure 4:**
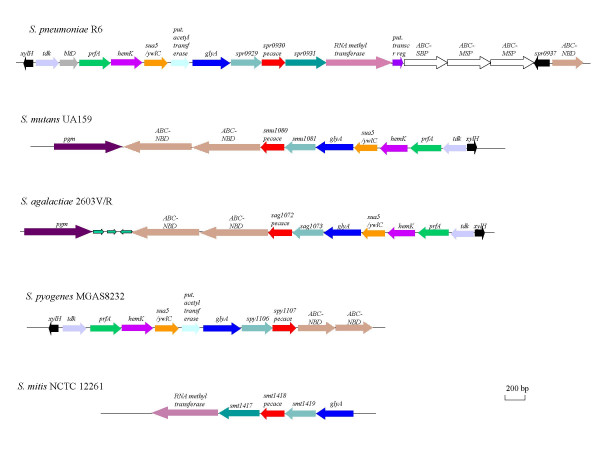
**Schematic representation of the gene cluster containing *pecace *in Streptococci species. **The coding regions and their direction of transcription are indicated by arrows. Gene names are given on top of the corresponding region.

The genomic organization of *pecace *genes has been analyzed in a variety of Gram-positive bacteria and a conserved distribution was observed in various streptococci species (Fig. 4). This feature indicates that genetic transfer of the whole cluster may have occured within the streptococci family, providing further evidence regarding the significant importance of the PECACE domains in bacterial physiology. However, the pneumococcal cluster is more related to the *S. mitis *one than to *S. mutans*, *S. agalactiae *and *S. pyogenes *ones, while clusters of the latter three species are related to each other. Genes located upstream and downstream of *pecace *are in some instances well characterized but the function of the corresponding proteins could not bring any clues about the role of PECACE, nor any evidence on *pecace *gene transcription. However, *pecace *is in all cases found in association with the same gene (whose locus name in *S. pneumoniae *is spr0929) but no information about the function of the protein encoded by this locus is available in databases. Transcriptional analysis of these two genes may bring informations about their potential co-regulation, a first stage in deciphering cellular function.

### Domain organization of proteins containing the PECACE domain

**Figure 5:**
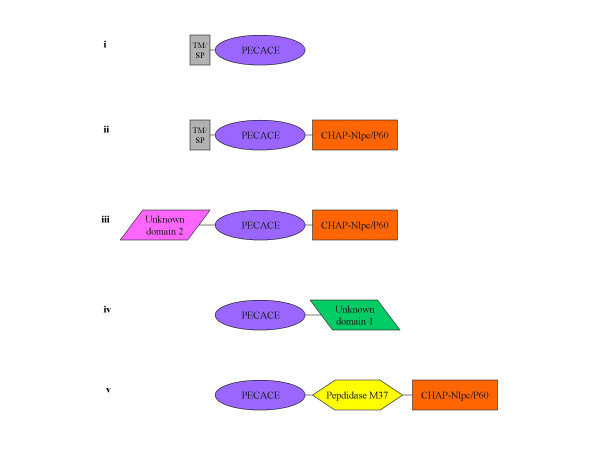
**Domain architecture of PECACE proteins. **The domain architecture of the proteins containing the PECACE domain was organized according to searches with NCBI Conserved Domain Search server against Pfam database: CHAP/NlpC-P60 (Pfam: PF05257/PF00877), M37 peptidase (Pfam: PF01551), unknown domain 1 (gi: 33355845) and unknown domain 2 (gi: 30021796). The size of the domains is not respected in these representations.

The PECACE domain is found in a large range of protein architectures, commonly associated with other peptidoglycan hydrolases, suggesting that these proteins have multiple peptidoglycan cleavages activities (Fig. 5). The identification of proteins displaying the PECACE domain was carried out using NCBI Conserved Domain Search and Pfam servers. In addition, prediction of membrane anchoring was performed with the DAS-Transmembrane Prediction server while extracellular secretion of the protein was deduced from the identification of a signal peptide.

PECACE-containing proteins appear to fall into 5 main categories (Fig. 5): (i) those which display no additional domain, (ii) CHAP-Nlpc/P60 as the associated group, (iii) CHAP-Nlpc/P60 and an unknown domain as associated groups, (iv) domains with no ascribed functions and finally (v) CHAP-Nlpc/P60 and M37 peptidase as associated groups.

The 19 proteins which contain only the PECACE domain belong to group (i) and harbor either a signal peptide or a transmembrane helix (as for the *S. pneumoniae *protein), leading in both cases to cell surface expression.

The CHAP-Nlpc/P60 domain is commonly associated with the PECACE domain in different modular organizations, namely in groups (ii), (iii), and (v) [18,19,27]. The CHAP domain has been recently described as a Cysteine, Histidine-dependent Amidohydrolase/Peptidase and it has been proposed to hydrolyse peptidoglycan containing γ-glutamyl [18,19]. Indeed, proteins such as *N*-acetylmuramyl-L-alanine amidase and D-alanyl-glycyl endopeptidase have been described as CHAP-containing enzymes [18,19]. However, while the substrate and the reaction mechanism have not been yet experimentally characterized for the CHAP domain, its role in peptidoglycan hydrolysis is inferred from its presence in multifunctional proteins recognizing peptidoglycan as substrate. Recently, hydrolytic activity of peptidoglycan has been attributed to the CHAP-containing protein PcsB in *S. pneumoniae *due to abnormal and uncontrolled cell wall synthesis at misplaced septa and formation of long cells in *pcsB *deleted mutant strains [20]. Proteins from group (ii) are expressed at the cell surface through a transmembrane anchor or are secreted, 12 members have been identified with this topology. Only one sequence (AAQ16265, gi:33355845) from *Enterococcus faecalis *BM4518 is part of the group (iii), and no function could be identified for the N-terminus domain preceding the PECACE domain. However the former domain is Lys-rich (14%) suggesting an electrostatic interaction with the peptidoglycan as proposed for *B. subtilis *endopeptidase [28]. Group (iv) is composed of an unique sequence from *B. cereus *ATCC 14579 (NP 833427, gi:30021796). Neither a signal peptide nor a transmembrane anchor have been detected. Furthermore, the domain of unknown function, which is different from the ones identified in groups (iii) and (v) is present in other multimodular proteins of *B. cereus*, in association with peptidoglycan hydrolysis enzymes. Finally, two sequences share the architecture defining the group (v) which harbor CHAP-Nlpc/P60 and Peptidase M37 domains [29]. Members of the Peptidase M37 family are generally glycylglycine endo-metallopeptidases; the archetypal member is the lysostaphin enzyme from *Staphylococcus *species which cleaves the pentaglycine bridge in the peptidoglycan [30]. One group (v) protein (NP 652875, gi:21392795) is encoded by *Bacillus anthracis *plasmid pXO1 and is required for synthesis of various anthrax toxin proteins [31]; this sequence has neither a signal peptide nor a transmembrane region. The second sequence of group (v) is located on *Bacillus cereus *ATCC 10987 plasmid pBc10987 (NP 982030, gi:44004362) and contains, in addition to CHAP-Nlpc/P60, Peptidase M37 and PECACE domain as well as an extra sequence to which no function has been attributed but with significant similarity with a *B. anthracis *plasmid pXO1 sequence (NP 652874, gi:21392794) [32].

## Conclusions

In summary, a new domain named PECACE, putatively involved in peptidoglycan cleavage has been identified in *S. pneumoniae*. The probable enzymatic activity deduced from the detailed analysis of the amino acid sequence suggests a LT-type or goose lyzosyme-type mechanism; we are currently characterising the enzymatic properties and cellular role of the PECACE domain from *S. pneumoniae*. This new putative pneumococcal peptidoglycan cleavage enzyme differs largely from the other hydrolases already identified in this bacteria. Indeed, LytA, LytB, LytC and CBPD proteins are all bound to the cell wall choline residues and thus expressed at the cell surface. The presence of a signal peptide within the amino acid sequence of PcsB suggests that it is either exposed on the cell surface or secreted. On the contrary, the pneumococcal NP358524 protein displaying the PECACE domain is embeded in the cytoplasmic membrane by a hydrophobic helix. The physiological role of this membranous peptidoglycan cleavage enzyme might differ from the other peptidoglycan hydrolysing enzymes.

Interestingly, the PECACE domain has only been found in Gram-positive bacteria. It is tempting to speculate that the multilayered structure of Gram-positive peptidoglycan relates to the PECACE putative activity.

The architecture of multimodular proteins containing the PECACE domain is another example of the pattern of multiple activities harbored by many peptidoglycan hydrolases, probably needed for the regulation of peptidoglycan metabolism. The release of new bacterial genomes sequences will probably add new members that will complete the five groups identified so far in this work and new groups could also emerge. Conversely, the functional characterization of the unknown domains mentioned in this work should now be easier, as their substrate, the peptidoglycan, is now identified.

## Methods

The non-redudant database of protein sequences (National center for Biotechnology Information, NIH, Bethesda) and whole bacterial genomes sequences [26] was searched using BLASP and PSI-BLAST programs  with (E) value threshold of 0.005 [33]. Multiple alignments were constructed with ClustalW program [34] followed by manual correction based on PSI-BLAST results. Protein fold recognition through 3D-profiles was searched using 3D-PSSM server [25]. Conserved (and degenerated) amino acid patterns was designed and searched against non-redudant database of protein sequences . Identification of domains associated with PECACE proteins was realized using NCBI Conserved Domain Search [35] and Pfam servers [36]. Finally, prediction of transmembrane anchor and secretory signal peptide were performed with DAS server  and SignalP-2.0 servers  respectively [37,38].

## Authors' contributions

OD conceived of the study and participated in the sequences alignment. EP carried out the sequences analysis and the writing of the manuscript with AMDG. TV coordinated the study. All authors read and approved the final manuscript.
